# Management of patients with posterior urethral valves “from the fetus to adolescence”: French national diagnostic and care protocol (NDCP)

**DOI:** 10.1186/s13023-025-03712-5

**Published:** 2025-05-12

**Authors:** Alaa El-Ghoneimi, Luke Harper, Ugo Maria Pierucci, Thomas Blanc, Jonathan Rosenblatt, Nicolas Sananes, Sophie Dreux, Marianne Alison, Fred Avni, Stéphane Decremer, Veronique Baudouin, Sayaka Oguchi, Dan Baruch, Pascale Rolland-Santan, Hedyeh Nadafi-Stoeffel, Cécile Bonnet, Annabel Paye-Jaouen, Eliane Raffet, Lise Natio, Berengere Desprez, Delphine Demede, Marc David Leclair, Matthieu Peycelon

**Affiliations:** 1 Department of Pediatric Surgery and Urology, National Reference Center for Rare Urinary Tract Malformations (CRMR MARVU), ERN eUROGEN, Robert-Debré University Hospital, APHP, GHU North, Université Paris Cité, 48, Boulevard Sérurier, 75019 Paris, France; 2 Department of Pediatric Surgery, Pellegrin Children’s Hospital, CHU Bordeaux, National Reference Center for Rare Urinary Tract Malformations (CRMR MARVU), Bordeaux, France; 3 Department of Pediatric Surgery, Buzzi Children’s Hospital, 20154 Milan, Italy; 4 Department of Pediatric Surgery and Urology, National Reference Center for Rare Urinary Tract Malformations (CRMR MARVU), Necker-Enfants Malades University Hospital, AP-HP, Université Paris Cité, Paris, France; 5 Department of Gynecology, Obstetrics and Prenatal Diagnosis, National Reference Center for Rare Urinary Tract Malformations (CRMR MARVU), Robert-Debré University Hospital, APHP, GHU North, Université Paris Cité, Paris, France; 6 Department of Gynecology and Obstetrics, Hôpital Américain de Paris, Neuilly-Sur-Seine, France; 7 Department of Prenatal Biochemistry, Hormonology and Biochemistry, DMU Biogem, National Reference Center for Rare Urinary Tract Malformations (CRMR MARVU), Robert-Debré University Hospital, APHP, GHU North, Université Paris Cité, Paris, France; 8 Department of Prenatal and Pediatric Imaging, National Reference Center for Rare Urinary Tract Malformations (CRMR MARVU), Robert-Debré University Hospital, APHP, GHU North, Université Paris Cité, Inserm U1141, NeuroDiderot, Paris, France; 9 Department of Imaging, Marie Curie Hospital, Charleroi, Belgium; 10 Department of Pediatric Nephrology, CHU de Toulouse - Hôpital Des Enfants, Toulouse, France; 11 Department of Pediatric Nephrology, National Reference Center for Rare Urinary Tract Malformations (CRMR MARVU), Robert-Debré University Hospital, APHP, GHU North, Université Paris Cité, Paris, France; 12 Cabinet Médical, Valenton, France; 13 Department of General Practice, School of Medecine, Université Paris Cite, Paris, France; 14 Cabinet Médical, Montpellier, France; 15 Department of Pediatric Surgery and Urology, National Reference Center for Rare Urinary Tract Malformations (CRMR MARVU), Hôpital Femmes – Mères – Enfants, Université Lyon 1, Bron, France; 16 Department of Pediatric Surgery and Urology, National Reference Center for Rare Urinary Tract Malformations (CRMR MARVU), CHU Nantes, Université de Nantes, Nantes, France; 17 UMR INSERM 1141 NEURODEV, Paris, France

## Abstract

Posterior urethral valves (PUV) are congenital anomalies characterized by the persistence of mucosal folds in the urethra, leading to various degrees of obstruction. They are the most common cause of lower urinary tract obstruction in fetuses and children, with a severe prognosis, as one-third of affected children develop end-stage renal disease before adulthood. The French National Diagnostic and Care Protocol (NDCP) aim to provide healthcare professionals with guidelines for the optimal diagnostic and therapeutic management of PUV from the fetal stage to adolescence. The guidelines emphasize early diagnosis through prenatal ultrasound and the importance of a multidisciplinary approach involving pediatric urologists, nephrologists, and other specialists. It outlines prenatal interventions such as vesico-amniotic shunting and postnatal surgical options like endoscopic valve ablation to alleviate obstruction and preserve renal function. Long-term follow-up is crucial for monitoring renal function, managing bladder dysfunction, and preventing complications such as urinary tract infections and chronic kidney disease. The guidelines also identify off-label pharmaceuticals and necessary specialty products not typically covered by insurance. By standardizing care pathways and promoting consistent, high-quality care, the guidelines aim to improve the prognosis and quality of life for children with PUV, setting a benchmark for managing this rare condition in pediatric urology.

## Introduction

Posterior urethral valves (PUV) are a congenital anomaly characterized by the abnormal persistence of mucosal folds in the lumen of the urethra resulting in various degrees of upstream obstruction [1]. They are the most common cause of lower urinary tract obstruction (LUTO) in the fetus and in children [2]. There is a very wide spectrum of severities and manifestations, ranging from mild forms in the prenatal period with ultrasound findings within the normal range, to a very critical picture of almost complete obstruction with early and severe antenatal ultrasound findings like those of urethral atresia [3]. In these critical cases, severe renal and bladder abnormalities may be seen during the fetal period. PUV may result in oligohydramnios or even anamnios. They may be associated with impaired lung development and neonatal lung function. PUV thus represent a neonatal emergency if the pregnancy is full term, and an indication for a termination in the most critical forms. Congenital anomalies of the urinary tract constitute about 15–21% of all congenital anomalies discovered at birth with a reported incidence of 1:250 to 1:1000 pregnancies [4]. According to the Parisian Congenital Malformation Registry, these congenital urinary tract anomalies represent approximately 17% of all malformations, varying between 15 and 20%, depending on the year [5]. The incidence of PUV ranges from 1:3,000 to 1:8,000 live births [6]. In specific populations, this incidence has decreased over the years where a prenatal diagnosis of severe forms can lead to termination of pregnancy [3]. The exact causes of PUV remain unknown and the embryological mechanism of this anomaly is not fully understood. It is hypothesized that PUVs are a result of disturbed embryonic development of the male urethra between the 9 th and 14 th gestational weeks (GW) related to migration of the Wolffian ducts. The inheritance of PUV is also poorly understood, and several genes and patterns of inheritance appear to be involved [3]. Antenatal obstruction results in both anatomical and functional alteration of the urinary tract, whose severity varies according to the degree of obstruction. The urinary tract may be affected [7]:

- The posterior urethra may be dilated and elongated, with a deformed *veru montanum* and ejaculatory ducts that are dilated in relation to an urethro-ejaculatory duct reflux.
- LUTO leads to increased bladder pressure, detrusor thickening, trabeculations and even the formation of bladder diverticula.
- The ureters can become dilated in various ways: either because of vesico-ureteral reflux (VUR), present in 50% of cases, which may be responsible for reflux nephropathy, from obstructive dysplasia or thickening of the bladder wall.
- Bilateral dilation of the upper urinary tract leads to impaired renal function and renal dysplasia beginning in embryonic life (tubulo-glomerular dysfunction).

## Methods

This manuscript is the official French National Diagnosis and Care Protocol (NDCP) guideline endorsed by the French National Authority for Health (Haute Autorité de Santé—HAS), designed to standardize management and care pathways for posterior urethral valves. It incorporates expert consensus from the 17 participating French centers and aligns international best-practice evidence. The objective of this NDCP is to provide healthcare professionals with a description of the current optimal diagnostic and therapeutic management and the care pathway for patients with PUV, from the fetus to adolescence. The elaboration of this protocol is coordinated by the National Reference Center (CRMR) for rare congenital urinary tract malformations (MARVU) and endorsed by the French HAS. The goal of this NDCP is to optimize and harmonize the management and follow-up protocols for this rare disease. It also identifies medications that are not currently approved by the European Medicines Agency (EMA). However, the French NDCP cannot take into consideration all specific cases, comorbidities or complications, therapeutic specificities or all hospital care protocols. It does not claim to cover all possible management approaches or to replace the physician’s individual responsibility to his patient. Nevertheless, this protocol describes the reference standard of care for a patient born with PUV from the fetus to adolescence. This NDCP has been drafted according to the “Method for drafting a national protocol for the diagnosis and care of rare diseases” published by the HAS in 2012 [8].

A systematic literature review was conducted to gather available data on PUV. The databases queried included general databases such as BDSP, Irdes, Refdoc, Medline, Embase, Thèses.fr, the National Library for Public Health, Google Scholar, Current Contents, and SciSearch. Additionally, specialized databases including EURONHEED, the Cochrane Library, and Prospero were explored. Governmental agencies such as the Haute Autorité de Santé (HAS) in France were also consulted, along with disease-specific websites like patient association pages, Orphanet, and therapeutic databases.

The search focused on studies published between 2000 and 2023, though key articles from expert opinions or prior working groups published before 2000 were also included when deemed essential. Publications in both English and French were considered.

A PICO-based methodology was employed to define the search strategy. Keywords used included posterior urethral valves and their variations such as lower urinary tract obstruction, bladder neck obstruction, and obstructive nephropathy. Diagnostic and therapeutic management terms included diagnostic, screening, detection, assessment, guidelines, best practices, differential diagnosis, genetic factors, histopathology, renal dysplasia, oligohydramnios, pulmonary hypoplasia, fetal MRI, ultrasound, voiding cystography, voiding cystourethrography, prognosis, biomarkers, congenital anomalies, kidney development, chronic kidney disease, and bladder dysfunction. Terms related to treatment and management strategies encompassed management, treatment, healthcare pathways, system care course, transition of care, transplantation, endoscopic resection using cold knife, diathermy, Bugbee, laser, bladder neck incision, circumcision, vesicostomy, urinary diversion, and antibiotic prophylaxis.

A total of 5,214 references were identified through database searches. After removing duplicates, 5,067 references remained. Following an initial screening, 4,569 references were excluded as irrelevant. Abstract evaluations were conducted on 498 references, of which 292 were excluded. Full-text evaluation for eligibility was performed on 206 articles, with 124 subsequently excluded. Ultimately, 82 references were retained for synthesis (**Prisma Flow-Chart**). This selection process followed the PRISMA (Preferred Reporting Items for Systematic Reviews and Meta-Analyses) guidelines, ensuring a rigorous and systematic approach to identifying and analyzing relevant literature.

The inclusion criteria encompassed studies published after 2000 that focused on PUV, its management, diagnosis, and patient follow-up. Review articles summarizing multiple references were considered when they provided comprehensive insights. Key references, essential guidelines, and relevant grey literature such as working group reports and expert consensus documents were also included.
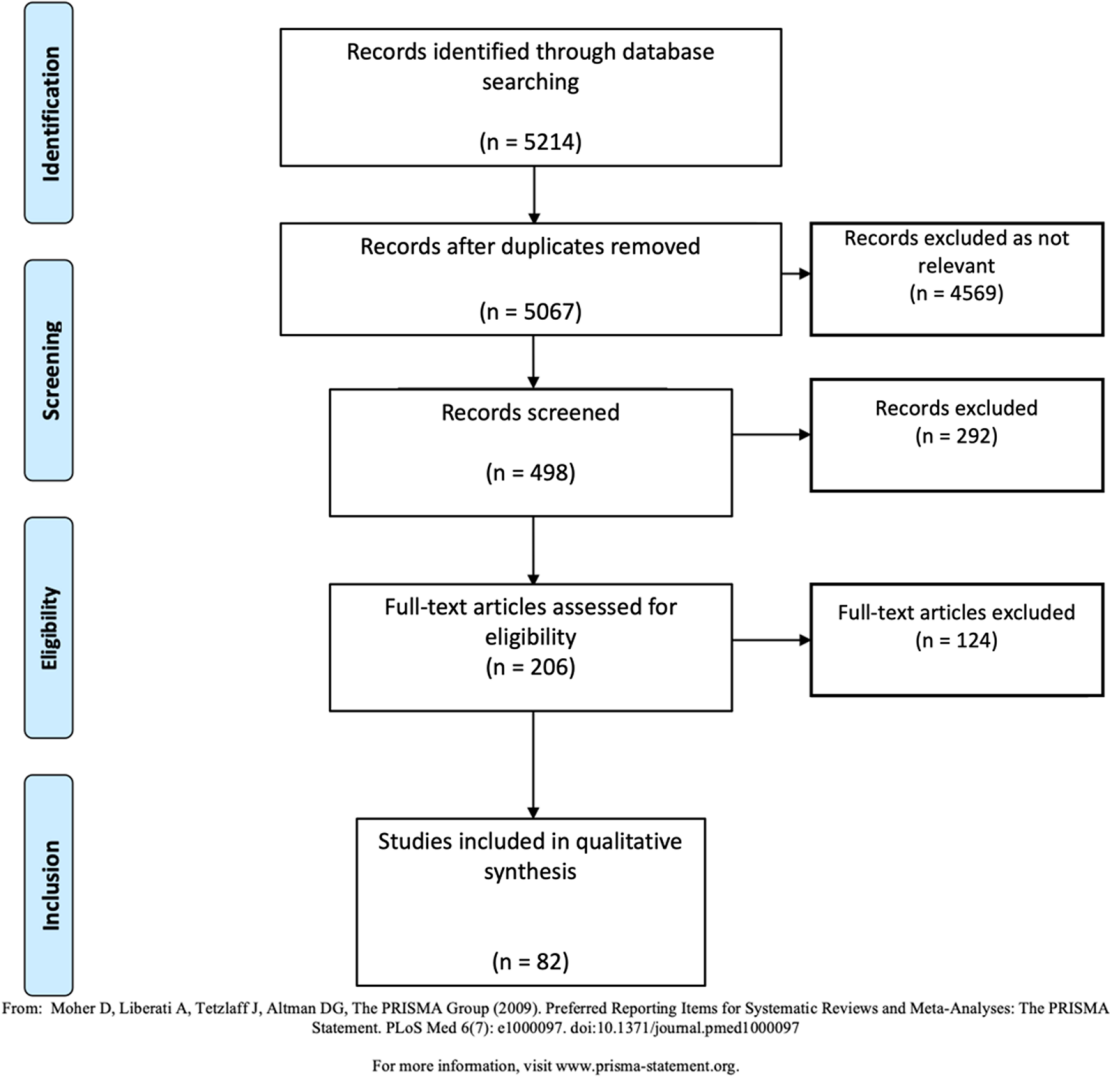


Prisma flow-chart

## Prenatal diagnosis

The role of prenatal ultrasound (US) screening is essential with a broad spectrum of symptoms. In the mildest cases, a prenatal diagnosis allows for optimal management and pediatric follow-up from birth. In the most severe cases with poor prognosis or with associated malformations, a termination may be discussed [3].

### Imaging

On prenatal ultrasound, the basic sign is a persistent and significant hydronephrosis (pelvic anteroposterior diameter (APD) > 10 mm). Several presentations of PUV can be observed:

- In the first trimester of pregnancy, the diagnosis is made in the presence of megacystis (> 3 cm) that may be associated with the presence of a dilation of the ureters and sometimes of the upper urinary tract, uni- or bilaterally. First trimester megacystis is not specific to PUV and may reveal many pathologies^9^. When aneuploidies have once been ruled out, two main differential diagnoses are possible but rare: urethral atresia and Prune Belly syndrome (PBS).
- During the second and third trimesters of pregnancy, ultrasound screening shows signs of LUTO with different degrees of urinary tract dilation.

In the most severe and classic form, there is a dilation of the upper urinary tract. Dilation of the posterior urethra is best analyzed by a sagittal view through a transperineal approach (when technically possible) showing a large bladder (> 5 cm high) with a crenelated and thickened wall that does not empty during successive examinations, bilateral ureteral dilatation, bilateral pelvic and caliceal dilation, and finally a hyperechogenic renal parenchyma, with frequent cortical or even medullary cysts [3, 10]. This classic form is usually associated with oligohydramnios, which may progress to anamnios over time. The diagnosis is usually clear when all these ultrasound criteria are present. An early diagnosis is associated with poor outcome [11]. However, this classical form is not the most common one and prenatal US can identify a non-specific hydronephrosis, which can change over the time towards a partial or complete LUTO. In particular, it should be noted that the diagnosis of PUV should not be excluded in the presence of a unilateral hydronephrosis in a male fetus. Thus, any obstructive uropathy in a male fetus should suggest the diagnosis. Based on baseline ultrasound, additional ultrasounds, sequential, if necessary, will look for signs that may add specificity to the diagnosis. It is most often a combination of arguments that suggests or confirms the diagnosis, but it is important to note that no prenatal ultrasound sign is pathognomonic of PUV. On the other hand, visualization of a normal urinary flow on color Doppler or in B mode ultrasound in the presence of an upper urinary tract dilation suggests differential diagnoses.

### Differential diagnosis

The differential diagnoses of prenatal PUV include all obstructive and non-obstructive lower urinary tract conditions that cause megacystis in boys. These mainly include VUR, PBS, urethral obstruction (secondary to atresia), or obstructive ureterocele. Other diagnoses include isolated physiological megacystis, neural tube defects or megacystis-microcolon intestinal hypoperistalsis syndrome (MMIHS) [12].

*In the first trimester* PBS is a congenital anomaly characterized by a dilated urinary tract, absent or hypoplastic abdominal wall muscles and undescended testes. On prenatal ultrasound, it is difficult to differentiate this syndrome from PUV. There is severe ureterohydronephrosis, megacystis with poor emptying, thickening of the bladder walls with possible enlargement of the proximal urethra. Signs of renal dysplasia may be present, with hyperechogenicity of the renal parenchyma, the presence of cysts, decreased cortico-medullary differentiation or oligohydramnios [13]. The presence of cryptorchidism and/or an irregular abdominal wall can help confirm the diagnosis. Urethral atresia is a closure or lack of opening of the urethra’s connection to the bladder. These anomalies cause early and severe signs in utero, with first trimester megacystis, anamnios from 17 weeks of gestation (WG) and a Potter sequence. Developmental abnormalities of the urorectal septum, primarily cloaca, are a rare differential diagnosis of first trimester forms. Cloaca in a male fetus is seen as megacystis in the first trimester, which may be very similar to a tight PUV or urethral atresia. The diagnosis can be made by urinary biochemistry with the detection of digestive enzymes in the bladder urine [14, 15].

*In the second and third trimester *The Megacystis Megaureter syndrome (MMS) is the main differential diagnosis. Reflux is related to a functional and/or anatomical abnormality of the VU junction. VUR may be suspected in the presence of thin-walled megacystis associated with fluctuating pelvic dilation, which may be permanent when the ureter is significantly dilated. In this condition, the bladder wall is thin and there is no dilation of the posterior urethra downstream from the bladder neck on the sagittal transperineal views. It is not possible to differentiate MMS from primary obstructive megaureter, as the ultrasound features are quite similar, and bladder size is not a sufficiently specific criterion. Thickening of the bladder walls is highly suggestive of a LUTO, whereas megacystis, especially in the 2nd trimester, also suggests a VUR and remains a very non-specific sign. The megalourethra is a result of lack of development of the erectile tissue and results in a dilation of the anterior urethra. The obstruction is more functional than anatomical. In rare cases, the presentation may be like that of PUV. MMIHS is a rare autosomal recessive congenital anomaly characterized by intestinal obstruction associated with megacystis. It is a genetic defect of the smooth muscle that can affect the bladder and the digestive tract. The absence of transit during the prenatal period does not confirm the diagnosis of functional microcolon. Thin-walled megacystis is most often seen, with or without urinary tract dilation, without renal dysplasia. The colon is poorly visualized on prenatal US due to the position of the bladder (functional microcolon is a postnatal sign). The amniotic fluid is usually normal [15]. Biochemical analysis of urine and amniotic fluid can support the diagnosis, which can be confirmed by a genetic analysis [15, 16]. Fetal T1-weighted MRI sequences can demonstrate the microcolon. In addition, LUTO with megacystis can be induced by a urethral prolapse of an ureterocele in connection with an upper moiety of a duplex system.

## Evaluation of the prognosis

### Fetal imaging

The phenotypic variability on ultrasound, with a wide spectrum of severities from one fetus to another and for the same fetus at different times in the pregnancy, complicates the prognosis when a diagnosis is suspected. Except for severe and very early forms, with a diagnosis made at the beginning of the 2nd trimester, determining the prognosis is a challenge based on ultrasound and an assessment of fetal renal function. Morris et al*.***,** in a meta-analysis by pooling data from 13 studies on 215 pregnancies with confirmed postnatal PUV, identified only three imaging criteria with prognostic value [11]:

- *Early suspicion or diagnosis* at less than 24 GW is a factor of a poor prognosis. Forms that appear earlier are probably associated with tighter stenosis and result in more severe renal lesions at birth. These data are more interesting for a specificity of over 80% than for their sensitivity of less than 50%
- *Oligohydramnios* is defined as an amniotic fluid index < 6 cm [17]. The amount of amniotic fluid reflects fetal diuresis. The clinical form may be associated with oligohydramnios depending on the severity of the lesions. Tight PUVs may have a mechanical impact on the amount of amniotic fluid. On the other hand, the absence of oligohydramnios does not exclude a diagnosis of PUV. Finally, oligohydramnios most often occurs in the 3rd trimester of pregnancy. Fetuses exposed to prolonged anamnios starting in the 2nd trimester may develop a Potter sequence with joint deformities (club feet, clenched/hyperflexed hands, arthrogryposis), characteristic facial dysmorphia with low-set ears, hypertelorism, flattened nose) and above all pulmonary hypoplasia [3], responsible for ventilatory difficulties with a high risk of perinatal death and can be objectively assessed on fetal MRI [3, 18]. In most cases, anamnios in the 3rd trimester or at the end of the 2nd trimester is not associated with pulmonary pathologies [19]. There is no argument for preterm delivery if late anamnios occurs in the 3rd trimester of pregnancy. In the study by Morris et al*.*, oligohydramnios had a sensitivity of 63% and specificity of 76% for predicting renal failure [11].

*The appearance of the renal parenchyma* is the second prognostic factor. Baseline ultrasound, preferably with high frequency probes, looks for decreased corticomedullary differentiation, hyperechoic parenchyma, and the presence of cortical cysts. The presence of cortical cysts and hyperechogenicity of the renal parenchyma are associated with long-term impaired renal function, and these patients are at increased risk of developing renal failure after birth [20]. In the study by Morris et al*.*, the sensitivity of this factor was 57% and the specificity 84% [11]. Renal parenchymal studies using high-frequency probes have become more common in recent years and improve the detection rate of renal parenchymal abnormalities and the presence of cortical cysts [21].

*The pop-off effect* corresponds to the rupture of the urinary tract, most often at the renal pelvis or a calyx, due the pressure upstream from the urethral obstacle. A urinoma is diagnosed on ultrasound by visualization of a perirenal fluid collection whose size varies. In some cases, bladder rupture may result in fetal ascites. When the ascites occurs in the presence of a known LUTO, the diagnosis is clear. In other cases, a biochemical study of the fetal ascites fluid may provide a diagnosis of urinary ascites and a suspected diagnosis of PUV. Results concerning the prognostic value of these urinary tract ruptures are conflicting [1, 22–25]. The pop-off effect hypothesis is that unilateral decompression of at least one of the two kidneys would preserve fetal renal function. However, the results of studies with relatively small numbers of patients are heterogeneous, and no clear conclusions may be drawn about the prognostic value of this pop-off effect. In conclusion, the important prognostic parameters are oligo-anamnios, hyperechoic kidneys and early diagnosis (at the 2nd trimester).

### Fetal karyotype

Associated and/or syndromic forms of the disease have a reserved prognosis from the outset and PUV worsens the prognosis of the urinary malformation itself. Fetal karyotype is recommended for suspected PUV and megacystis. Aneuploidy may be found in 5–25% of cases and, the greater the number of other malformations, the greater the risk of aneuploidy [2, 26]. The prognosis for isolated forms is based on the amount of amniotic fluid, the appearance of the renal parenchyma and the term at diagnosis. Assessment of renal function by biochemistry provides additional prognostic criteria.

### Fetal biochemistry

*Fetal biochemistry tests in PUV* The goal of fetal renal function assessment is to distinguish severe cases from those with a better prognosis and to determine the management pathway. These tests are only performed if an anomaly is detected on ultrasound and can confirm a poor prognosis suggested by US. A multidisciplinary discussion based on imaging and fetal biochemistry results helps in determining the optimal management pathway and parenteral counselling: (such as a vesico-amniotic shunt for example), to have a premature birth (rare indication), to prepare postnatal management [27]. The indications for the antenatal assessment of renal function are as follows [28]:

- Poor progression of ultrasound features
- Assessment before possible in utero treatment
- Assessment before possible termination of pregnancy when a poor prognosis is suspected
- In case of a parental request.

Maternal blood and amniotic fluid cannot be used to assess fetal renal function. Although several biochemical markers of amniotic fluid have been studied in the literature, none of them currently provides a reliable answer [29, 30]. However, advances in proteomic techniques offer hope that the analysis of amniotic fluid can soon be used to predict fetal renal function [31–34].

*Fetal Urine* If dilation of the urinary tract is significant and decompression appears to be beneficial, fetal urine is the sample of choice. Fetal urine is collected at the dilated pelvis and/or bladder, preferable from the least dilated pelvis and bladder. Sampling errors can result in poor assessment of renal function. A mistakenly poor prognosis can be made if the sample is taken from an ureterocele (the non-functioning kidney), or from a renal cyst [27]. Strict biochemical interpretation criteria must be applied, and the test should only be sent to accredited laboratories. The presence of secretions from amniotic fluid contamination may distort bladder sample results, and fetal urine is not suitable to assess renal function in this case. Fetal urine sampling can be performed earlier than fetal blood sampling allowing the selection of fetuses for shunt placement or in utero surgery. Several biochemical markers in fetal urine are studied simultaneously: total protein, β2-microglobulin, sodium, chlorine, glucose, calcium and phosphorus. Although these are classical markers, technical adaptations must be made because the values in fetal urine are different from those in postnatal urine. Normal fetal urine values have been established retrospectively in cohorts with known long-term outcomes [20, 35]. The sensitivities and specificities of the separate urine markers are, respectively: 80.6% and 89% for β2-microglobulin, 61.3% and 100% for sodium and 64.5% and 100% for calcium [36, 37]. Longitudinal cohort studies have identified the best predictors of long-term renal function. Indeed, in most studies, fetal renal function predicts postnatal renal function at two years of age. However, at least one study has correlated the results of fetal urinalysis with the progression of renal function after 10 years of age. In that study, multivariate analysis of fetal urine parameters predicted renal function after 10 years with a sensitivity and specificity of 93% and 71%, respectively [38]. To avoid stagnation, which could modify urinary marker values and results in a worse biochemical diagnosis than reality, sequential puncture of the fetal urine can be performed, often on two consecutive days. However, the iatrogenic risk must be weighed against the benefit of these repeated punctures [39]. Fetal urine sampling is also used to make a differential diagnosis with MMIHS [15, 16].

*Fetal Blood* Fetal blood collection (from the umbilical cord) can be performed after 20 GW. Fetal blood must not be contaminated with maternal blood or amniotic fluid. Fetal serum creatinine is not a good marker of renal function because they reflect those of the mother and not fetal renal function. Different markers have been tested with encouraging results: β2-microglobulin predicts renal function with 94.3% sensitivity and 89.5% specificity. Cystatin C is less sensitive (60.6%) and less specific (70.6%), and the performance of α1-microglobulin is disappointing (82.8% sensitivity but only 47.3% specificity). The cystatin C assay is technically more challenging and is therefore not used in clinical practice. β2-microglobulin is the only marker used as it is well correlated with postnatal renal function. Nevertheless, this correlation has only been demonstrated in obstructive uropathies such as PUV, renal dysplasia and renal hypoplasia. If the result of the fetal blood β2-microglobulin assay is too close to the grey zone (5 mg/L), a second sample can be taken at least 15 days later to determine whether fetal renal function has worsened, particularly if there is a change in ultrasound criteria. One study showed that, for these difficult cases, both sensitivity and specificity improved with a 2nd sample (96.4% versus 64.3% and 85.7% versus 78.6%, respectively) [40].

## Fetal procedures

Prenatal management of LUTO involves restoring bladder emptying. The rationale is that by relieving the hyperpressure on the urinary tract, renal function can be maintained. Recent data on echo-histological correlations support this hypothesis [41]. There are three main therapeutic approaches:

- Ultrasound-guided transparietal percutaneous placement of a vesico-amniotic shunt.
- Fetal cystoscopy with antegrade section of the valve.
- Ultrasound-guided transurethral balloon dilation.

To date, only one randomized study has evaluated antenatal therapy, investigating the value of vesico-amniotic shunt [42]. Inclusion criteria were the presence of an enlarged bladder with dilated posterior urethra, dilated urinary tract and cystic renal parenchyma. Patients could be included regardless of the amount of amniotic fluid, with no gestational age limit and without any biochemical analysis of fetal renal function. The study was prematurely stopped after the inclusion of 31 of the 150 planned patients due to a lack of recruitment. The statistical analysis concluded that the probability that vesico-amniotic shunt would improve neonatal survival was 86%. There was no improvement in renal function and the overall prognosis was poor in both groups. In addition, among the 15 patients with a vesico-amniotic drain, three cases of premature rupture of the membranes and four cases of drain displacement or obstruction were reported. Finally, it should be noted that in addition to the cases of PUV, eight cases of urethral atresia were included in this series. This study raises the questions of the distinction between PUV and other causes of LUTO, patient selection (especially in relation to kidney appearance, the amount of amniotic fluid, the gestational age, and the biochemical assessment of fetal renal function), and finally, the relevance of prenatal intervention for LUTO. Patient selection is also a crucial issue because antenatal procedure may not be relevant in mild LUTO (where the risks of treatment, including the premature rupture of membranes, outweigh the expected benefits), or in severe LUTO (where the benefit is too uncertain). Although several classifications or predictive scores have been designed to define postnatal renal function, none have been prospectively evaluated compared to a control group and therefore cannot be recommended [43, 44]. Finally, two meta-analyses have evaluated the value of vesico-amniotic shunt in patients with LUTO and have found an improvement in perinatal survival but discordant results for renal function [45, 46]. The major biases of the studies were that they were observational, usually retrospective and with control groups that are comparable to the intervention group. Moreover, combining these data is questionable because patient selection was heterogeneous from one series to another. Several studies have evaluated the value of fetal cystoscopy [47–50]. [51]. A multicenter series by Sananes et al. involving 50 fetal cystoscopies, of which 23 involved laser ablation, demonstrating a promising survival rate of approximately 60.9% [47]. Despite these encouraging outcomes, robust evidence comparing the superiority of fetal cystoscopy over vesico-amniotic shunting remains insufficient. Significant methodological limitations, including the lack of randomized controlled trials, small patient cohorts, and variable inclusion criteria, preclude definitive conclusions. Nevertheless, fetal cystoscopy, particularly utilizing laser ablation for obstructive valves, may offer diagnostic and physiological advantages by enabling etiological clarification and restoring bladder emptying [50]. However, technical challenges and associated risks, such as fistula formation, should be carefully considered [52]. Ultrasound-guided transurethral balloon dilation has also been described; however, available data primarily consist of limited case series, restricting definitive evaluation of its efficacy and safety profile [51]. Amnioinfusion has additionally been proposed as a therapeutic measure to normalize amniotic fluid volume and mitigate pulmonary hypoplasia risk. Yet, current evidence supporting its routine clinical use is limited and inconclusive, necessitating further well-designed studies to validate its clinical utility [53].

## Postnatal diagnosis and assessment

### Postnatal clinical assessment

The clinical examination of the newborn with a prenatal diagnosis of uropathy is systematic and looks for lumbar contact (in case of significant renal dilation), urinary retention, an anomaly of the external genitalia (hypospadias, undescended testicle) and aplasia of the abdominal wall muscles. An abnormal urine stream may be observed but it is inconstant and difficult to evaluate for a newborn. More rarely, palpation of a large kidney may suggest a LUTO [3]. The initial physical examination of the infant may reveal diffuse edema. The abdomen may be distended due to urinary ascites, bladder distension or secondary to urinary tract dilation (severe uni- or bilateral pelvic dilation, acute urinary retention) [3].

Subsequently, analysis of the antenatal ultrasound images will determine whether an urgent imaging assessment is required, and the usefulness of early antibiotic prophylaxis [11]. The most severely affected patients may present with respiratory distress during the neonatal period.

If the diagnosis of PUV was not suspected prenatally, a urinary tract infection (UTI) may develop postnatally, typically presenting with nonspecific symptoms such as abdominal pain or discomfort, hypotonia, poor weight gain, and feeding refusal. These UTIs can be severe, potentially leading to sepsis, characterized by systemic inflammatory response, instability in vital signs, and significant clinical deterioration. Moreover, severe UTIs in neonates with PUV can be complicated by transient renal tubular dysfunction, clinically presenting as pseudo-hypoaldosteronism (hyponatremia, hyperkalemia, metabolic acidosis, and elevated plasma aldosterone levels due to renal tubular resistance) [54]. This electrolyte imbalance can exacerbate clinical deterioration and requires careful management [54].

Clinical monitoring, including urine output and blood pressure, is crucial. Neonates must be weighed especially after PUV section, and this should be done every 12 h during hospitalization [3].

### Postnatal biological assessment

The first few days after birth, an initial assessment of the biological status of a newborn with PUV is usually misleading because of the residual effects of maternal placenta-mediated renal function. Initial blood creatinine and nitrogen values are usually like those of the mother, and it can take up to three days for serum values to accurately reflect the newborn’s renal function [3]. The frequency of monitoring of serum creatinine, blood nitrogen and electrolytes depends on the clinical status [3].

Three categories of postnatal renal function may be determined by biochemistry: good, very poor, and intermediate. Currently, serum creatinine is the most widely used marker to assess renal function. Renal function may be assessed with the nadir creatinine. The nadir corresponds to the lowest creatinine level during the 1 st year of life. Some authors agree that a nadir value above 0.99 mg/dL is predictive of poor renal function. In the general population, this value is reached at the 1 st or 2nd month of life. Patients with PUV reach this level at an average of five months of age. Children must be tested regularly during the first year of life to determine this value. The 0.99 mg/dL threshold is predictive of chronic kidney disease (CKD) stage 3 or higher at 2 years of age. An increase of more than 0.29 mg/dL is predictive of CKD stage 2 or higher at 5 years of age in patients who have not had a prenatal urological intervention [10]. Assessment of renal function (BUN and serum creatinine), blood and urine chemistries and urine culture, together with measurement of urine osmolality, should be systematically done. Proteinuria and microalbuminuria are markers of CKD but are rarely present in PUV and congenital anomalies of the kidney and urinary tract (CAKUT), except in cases of major nephron reduction or additional pathology. They are therefore not a reliable indication of renal function.

. If serum creatinine is 0.39 mg/dL at the age of two years, renal function can be considered normal. When the creatinine is above 0.85 mg/dL at the age of two, renal function is considered to be abnormal. No definitive conclusions regarding renal function can be drawn when creatinine levels range between 0.39 and 0.85 mg/dL at the age of two years; therefore, continuous follow-up is necessary to accurately determine renal status [55].

### Postnatal imaging

**Fig. 1:**
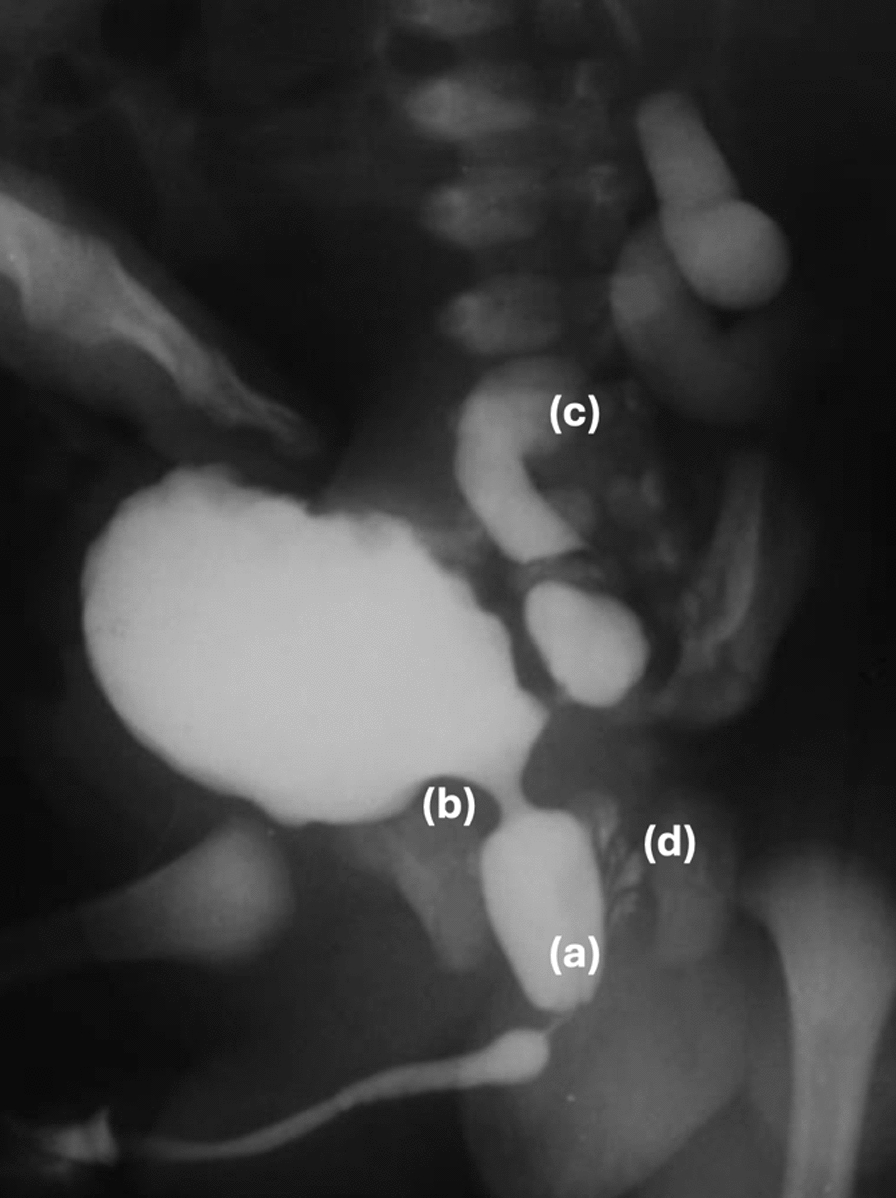
Suprapubic VCUG in a newborn with suspected antenatal PUV. Dilation of the posterior urethra **a**, hypertrophy of the bladder neck **b**, VUR with ureteral dilation **c**, and reflux into the ejaculatory ducts **d**

An early neonatal ultrasound scan should be systematically performed if there is antenatal suspicion of PUV. Results may show an enlarged bladder with a thickened wall, associated with dilation of the posterior urethra. This is more easily seen during micturition on a sagittal median section through the transperineal approach with a high frequency probe. On the other hand, in some cases, imaging may reveal a small, retracted bladder with a very thick wall. Ultrasound assesses the degree of dilation of the upper urinary tract and the extent of parenchymal lesions is the renal cortex. This examination is the basis for future examinations [28]. When the diagnosis of PUV is suspected on ultrasound, a voiding uretrocystography (VCUG) should be rapidly performed to confirm the diagnosis and excludes any differential diagnoses [28]. VCUG must follow strict and rigorous aseptic rules. The choice of the suprapubic or retrograde approach depends on the usual practices of the pediatric radiologists. The best angle for the diagnosis is a three-quarter or a profile view and centered on the bladder and urethra. The diagnosis of PUV is based on visualization of dilation and elongation of the posterior urethra with a dome-shaped appearance. The greater the obstruction, the thinner the anterior urethra appears. The bladder is trabeculated with a thick wall with diverticula. The bladder neck is more often abnormally hypertrophic. The post-voiding phase often shows incomplete emptying, hypertrophy of the bladder neck and reflux into the ejaculatory ducts (Fig. 1). In 40–50% of cases, a variable grade of VUR and upper tract dilation are present. VUR is often high-grade, usually bilateral but sometimes asymmetric [30].

### Delay diagnosis after the neonatal period

With the systematic antenatal diagnosis, a diagnosis of PUV is increasingly suspected before birth. When PUV is diagnosed after the neonatal period, the child usually presents with UTIs, sometimes pain or voiding disorders and acute renal failure. Therefore, any symptoms of LUTO in boys, including recurrent UTIs, dysuria, urinary retention, bladder distension, gross hematuria or impaired renal function should suggest PUV [3]. PUV is suggested on ultrasound, when it shows an enlarge bladder and dilation of the posterior urethra. On VCUG, dilation of the posterior urethra is confirmed. After the neonatal period, when there is a suspected LUTO, PUV should be differentiated from other possible diagnoses [3]:

- *High grade VUR* This differential diagnosis is sometimes the consequence of a prenatal non-neurological LUTO, with abnormal shape of the posterior urethra (dilation of the posterior urethra, impression of a sudden change in the caliber of the urethra) [56].
- *Neuropathic bladder* It should be considered if there is an abnormality of the sacrum. Voiding image confirms any urethral caliber abnormalities [57].
- *Penile urethral diverticulum* A rare anomaly primarily seen in infants, presenting with a ventral urethral bulge during voiding [58].
- *Anterior urethral valves* These are rare. The obstruction is in the anterior urethra and causes urethral dilation upstream. Diagnosis is based on VCUG and endoscopy. Management is identical to that of PUV [59].
- *Stenosis of the urethral meatus* Congenital stenosis is very rare. Most cases of narrowed meatus are seen in children circumcised in the first few weeks of life. The diagnosis suspected on clinical examination. When potty-trained, uroflowmetry may help make the diagnosis [59].
- *An obstructive polyp of the veru montanum* Malformation presents as an oblong subtraction image appended to the veru montanum and moving into the urethra [60].
- *Syringocele of Cowper’s duct* May present with lower urinary tract symptoms and is diagnosed with VCUG or urethroscopy [61].

## Neonatal surgical management

Postnatal management should occur in a specialized center, in a level 3 maternity hospital. As soon as the diagnosis of PUV is confirmed by VCUG, surgical management is scheduled. The main goal of surgical treatment is to remove the lower urinary tract obstruction [62]. Two surgical procedures are available: endoscopic valve ablation or urinary diversion.

### Neonatal endoscopic valve ablation

Thanks to the prenatal diagnosis, endoscopic valve ablation can be performed early, within 48 h after birth, and when the newborn is clinically stable [3]. This procedure, which involves endoscopic incision of the valves, is the surgical option of choice for any newborn with PUV. The aim of treatment is to release the urethral obstruction and allow normal cyclic bladder function alternating filling and emptying of the bladder. With the miniaturization of endoscopic instruments (< 2 mm), newborns weighing > 2,000 g at birth can now be treated with this technique. After introducing the endoscope and visualizing the valve, several methods of valve section have been described in the literature: 1) endoscopic ablation with a cold knife; 2) endoscopic fulguration by electrocoagulation such as the Bugbee electrode or diathermic loop, 3) endoscopic fulguration with a YAG laser. After incision, a urethral catheter may or may not be placed for 24 to 48 h depending on the pediatric urology department’s practices. Complications following endoscopic management of children born with PUV (stricture, retention, extravasation, hematuria, anuria), occur in between 5 and 25% of patients [63]. Post-operative urethral strictures are extensively described in the literature [64–66]. The rate is between 0 and 25%, higher after the use of electrocoagulation (8 to 25%) [64–66]. To minimize the risk of iatrogenic urethral stenosis, cold knife section is generally preferred over electrocoagulation [66, 67]. Although polyuria (or post-obstructive diuresis) is a common complication in boys after the removal of the obstruction, its incidence and duration are not well described in the literature. It is thought to affect one third of boys with a prenatal diagnosis. This risk of post-obstructive diuresis decreases with the age at diagnosis: children diagnosed later after birth are less likely to develop this complication [68].

### Neonatal urinary diversion

If the newborn is too small and weighs < 2,000 g, there are several options depending on the child’s weight. Bladder drainage is mandatory. Usually, a diversion is performed that may involve the upper urinary tract (pyelostomy/nephrostomy and ureterostomy) or the lower urinary tract (vesicostomy). This procedure, which is a subject of debate in the literature, is considered in the presence of persistently impaired renal function, increasing dilation of the upper urinary tract, severe urinary tract infection or whenever the endoscope cannot be introduced into the child’s urethra [62]. They are usually performed either during the neonatal period (before valve section) or during follow-up. At present, this option is indicated as a temporary solution to avoid deterioration of bladder function secondary to the absence of bladder cycling.

*Vesicostomy* is the surgical diversion technique of choice [69]. It is a temporary treatment to preserve renal function and renal growth until valve section can be performed. The main disadvantage of vesicostomy is that bladder cycling is less efficient than fully cycling bladder after valves section.

*Ureterostomy* Sober’s cutaneous ureterostomy is considered in case of unsuccessful drainage by vesicostomy or as an alternative to vesicostomy. Depending on the type of ureterostomy (terminal, lateral or Sober), Sober or lateral diversion, urine can flow from the kidney while preserving at least part of bladder irrigation compared to terminal ureterostomy. The Sober urinary diversion also avoids bladder surgery when it is time to close the diversion [70].

*Pyelostomy or nephrostomy* may be indicated in selected cases of urinary tract infection complicated by obstructive uropathy or pyonephrosis, serving as a temporary urinary diversion to relieve obstruction and preserve renal function [71].

## Neonatal management by pediatric nephrologists

Optimal management by a pediatric nephrologist, accompanied by a dietician is essential from birth because of the frequent electrolyte imbalance and the risk of dehydration. Management can temporarily slow progression towards end-stage renal disease (ESRD) but cannot totally prevent it in the case of renal dysplasia. The main goal of management is to ensure optimal weight gain and growth, which implies both appropriate nutritional intake and a good acid–base and electrolyte balance in the blood. Indeed, chronic dehydration and/or acidosis have a negative impact on growth. Nutritional status is particularly important because dietary deficiencies are detrimental to neurocognitive development and the subsequent function of other organs [72]. Whatever the level of renal insufficiency, and even in its absence, the initial risk is of electrolyte imbalance due to tubular damage generating a loss of sodium and disorders of urine concentration and dilution. It therefore necessary to monitor fluid balances from birth by measuring weight every 12 h at least to avoid dehydration. This hyperdiuresis may be increased in the first few days after bladder drainage, resulting in a post-obstructive diuresis. In the event of weight loss, water and sodium supplements are provided (from 2 to more than 10 mmol/kg/day) and adapted on a case-by-case basis. Acidosis is also frequent and must be treated by sodium bicarbonate supplements to maintain an alkaline reserve of more than 22 mmol/L. Water intake should be calculated and adapted to the disorders of urinary concentration and dilution, considering the osmole contribution of milk and the additional contributions of bicarbonates and sodium chloride. The level of glomerular filtration cannot be assessed at birth and there is no point in measuring creatinine levels during the first 24 h of life, as these only reflect the mother’s levels. The level of renal insufficiency can only be estimated in the following weeks after the valve has been resected. Classically, creatinine levels rise during renal failure until they stabilize in the first two weeks, and then glomerular filtration may improve over the following months as glomerular filtration matures physiologically. The need for dialysis is very rare in the first days of life because diuresis is usually preserved. Breastmilk is preferred than formula because it is lower in osmoles, but it may need to be supplemented to increase its nutritional value. If formula is used, formula milk for newborns and very young infants should be used in the first six months in infants with renal failure (there are formulas with less phosphorus and potassium), to control protein intake. When renal failure is severe, caloric supplementation is often needed based on a continuous nightly administration of enteral nutrition due to secondary anorexia and significant electrolyte imbalance (in practice almost obligatory once serum creatinine is > 1.69 mg/dL). Polyuria and salt loss may sometimes require a gastric tube for hydration and enteral nutrition. Excessively rapid and extreme weight loss and dehydration worsen renal function and should be avoided. In the most severe cases complicated by early oral disorders, a gastrostomy may be discussed later [72].

## Follow-up in childhood by a multidisciplinary team

A multidisciplinary approach is essential at each stage and age to optimize follow-up and to provide parents and healthcare providers with as much information as possible. The management of patients with PUV during childhood and adolescence is essential and may affect the long-term development of CKD. Regular assessment of voiding habits, the occurrence of fUTIs, nephrological follow-up, compliance with medication and voiding education are essential. The age at diagnosis, and the nadir of serum creatinine in the first year of life, the presence of a UTI or bladder dysfunction and the number of fUTIs are factors that have been identified as having a poor prognosis on renal function [73]. The aim is to minimize the risk of bladder dysfunction and maintain good long-term renal function. The need for a focused care pathway during adolescence and accompanied transfer to adult urology and nephrology services are necessary to maintain renal function and quality of life in adulthood [74].

**Fig. 2:**
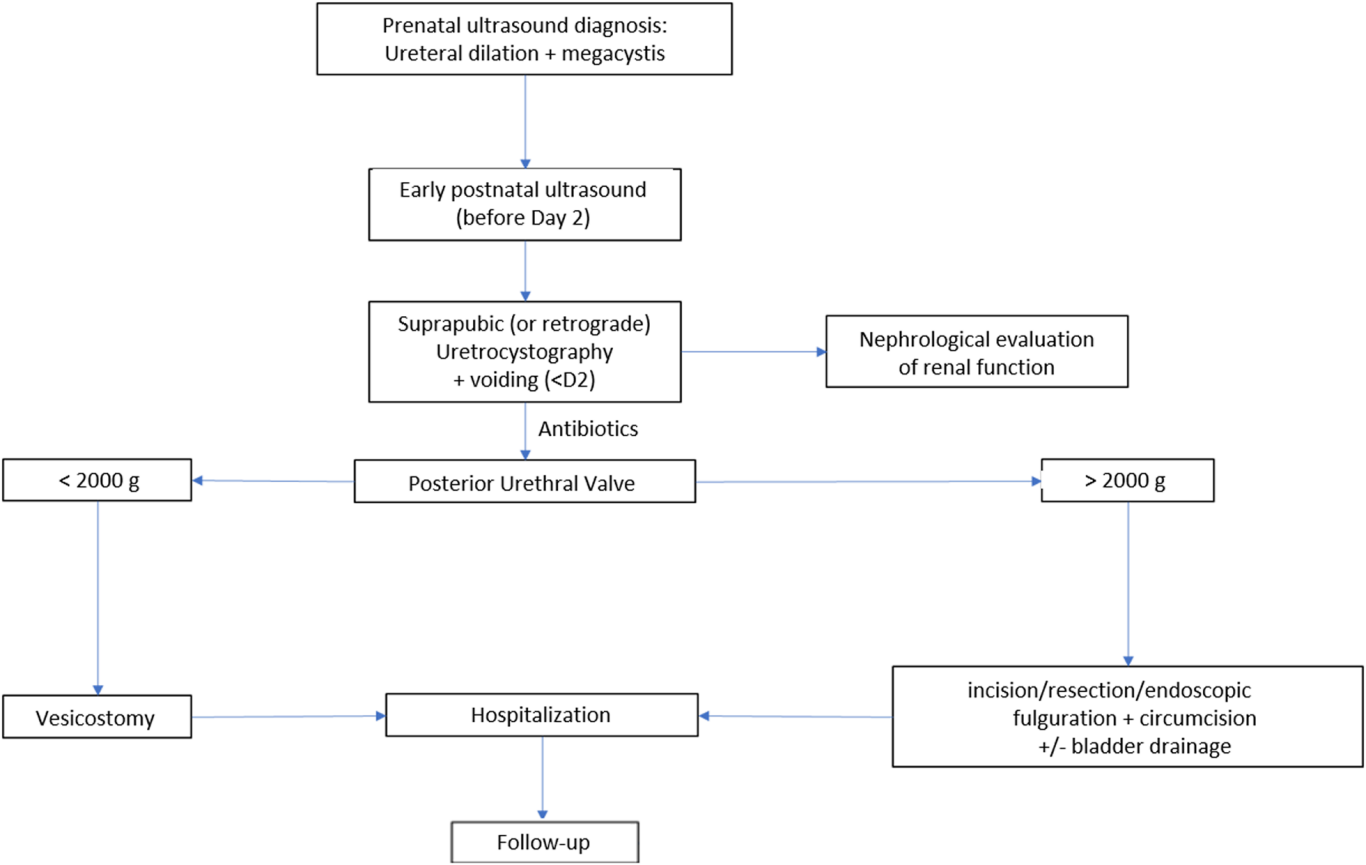
Algorithm for the management of PUV at birth

**Fig. 3:**
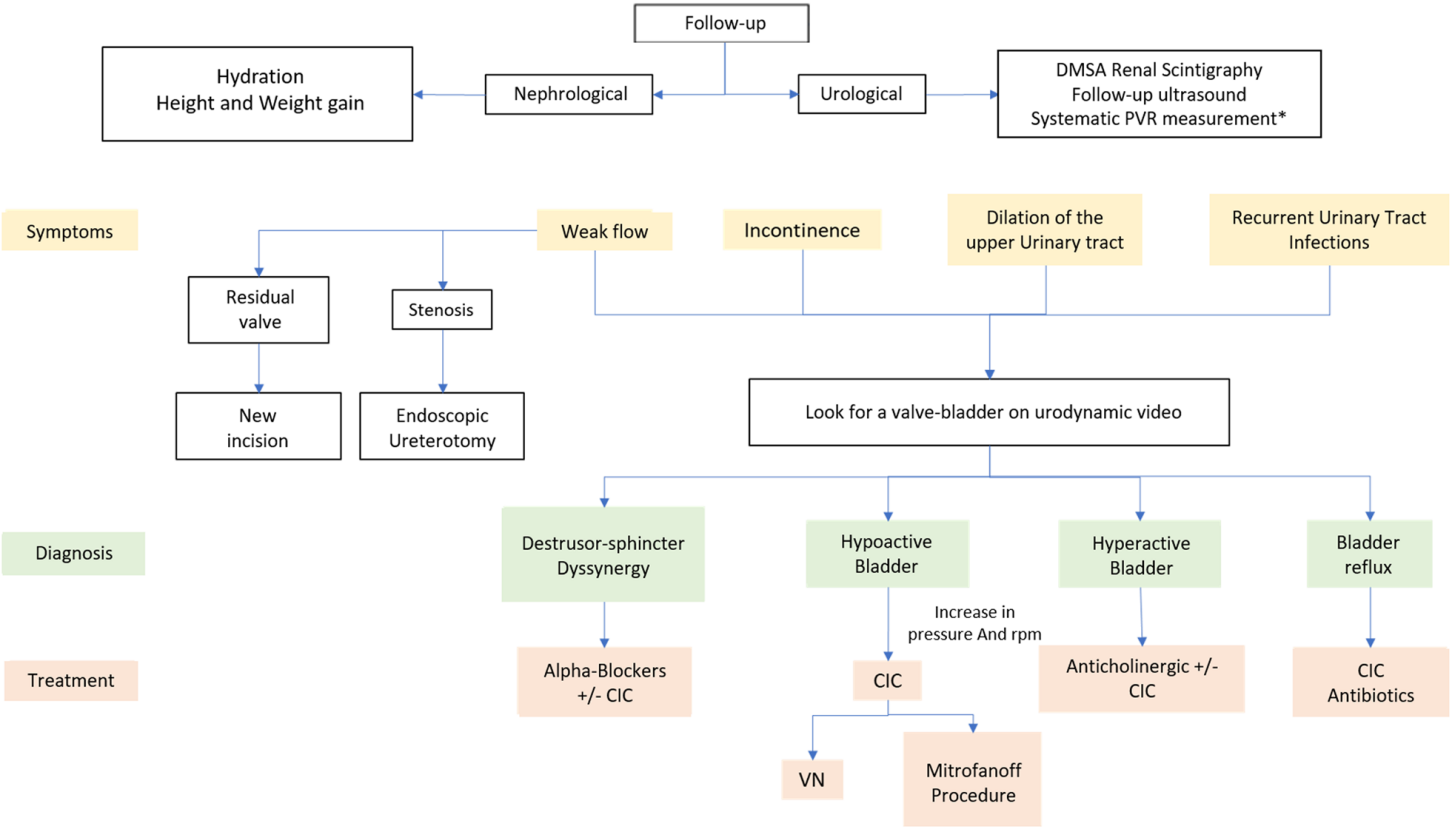
Algorithm for monitoring PUVs

### Role of imaging in follow-up

Routine PUV monitoring includes ultrasound of the urinary tract to follow the progression of dilations and the appearance of the bladder. Ultrasound is important in the follow-up of valve section and can be performed monthly at first then less frequently according to the findings. A VCUG is sometimes ordered in the follow-up after the valve resection in case of poor clinical or ultrasound progress [28]. The role of imaging, in particular ultrasound, in the follow-up of patients treated for PUV is mainly to confirm renal growth and the progression of anomalies noted at birth. Renal ultrasound is the first-line radiological examination in the event of complications (urinary tract infections, lithiasis, increased renal failure, etc.) [28]. The radiologist will then pay particular attention to kidney volume (related on charts to the child’s weight or age), kidney echostructure (appearance of cortico-medullary differentiation, dysplasia), the presence, number and size of cysts in the renal parenchyma, the cortical index (ideally at the upper, middle and lower poles), dilation of the urinary tract (calyces, pelvic, ureters), and finally the appearance of the bladder (thickness and regularity of the walls, measurement of a PVR). Follow-up ultrasound should be performed annually, and more frequently in the event of complications such as pyelonephritis, lithiasis or deteriorating renal function. Dimercaptosuccinic acid (DMSA) renal scintigraphy is used to assess relative renal function. It can also be used to search for cortical scars on the kidney. Above all, it has prognostic value. Unlike unilateral uropathy, where the affected kidney is compared to the healthy kidney, in PUV, renal involvement on scintigraphy, when it exists, is bilateral. Nevertheless, the appearance of new cortical notches or increased global heterogeneity can help make therapeutic decisions. This examination can first be performed during the neonatal period (around six months) to have baseline results, and then according to the clinical or biological elements suggesting a negative progression. Although cystosonography is very useful to monitor reflux associated with PUV, this depends on the experience in the centers. The advantages of this method include no radiation, continuous dynamic nature, and high sensitivity [75].

### Nephrological follow-up in childhood

Nephrological monitoring should be associated with monitoring of bladder function. Renal failure is the most feared long-term complication. Progression to CKD depends primarily on the severity of renal dysplasia, but also on neonatal management and, in childhood, on monitoring of bladder dysfunction, the number of febrile UTIs, and the presence of proteinuria. The goals of monitoring include: preventing and treating complications of CKD; slowing the progression of CKD; educating the patient and parents on the monitoring and treatment of CKD, and; anticipating renal replacement therapies (dialysis) and renal transplantation [72]. This follow-up is managed by a pediatric nephrologist, a pediatric urologist and a GP. Nephrological follow-up is essential, even if serum creatinine levels are normal, because this does not guarantee normal long-term GFR and tubular functions. Nephron reduction may be revealed late in childhood and adolescence, usually initially by the development of pathological microalbuminuria, then renal failure. The patient should be monitored clinically (growth with height and weight gain, blood pressure) and biologically. Serum creatinine and the microalbuminuria/creatinuria ratio should be regularly monitored (Box 1).

**Box 1:** Nephrological assessment at each consultation

Weight
Size
Blood pressure
Blood test: chemistry and bicarbonates, BUN and creatinine (± serum cystatin C) with estimation of GFR by simplified or composite Schwartz formula (before 15 years)
Phosphocalcium balance
Proteinuria, microalbuminuria and creatininuria on one voiding once a year after toilet training

Impaired renal function is often associated with fluid and electrolyte disorders that are detrimental to the health of the child. The most frequent disorders are hydrosodic depletion and acidosis related to polyuria and renal parenchymal damage. As mentioned earlier, these disorders may be present even in the absence of reduced glomerular filtration.

The child should be monitored regularly by the pediatric nephrologist to detect and treat the usual adverse consequences of CKD as early as possible. (Box 2).

**Box 2:** The management in the NDCP on chronic kidney disease in children

1	Maintain height and head circumference growth according to age-related curves, which implies:	
		Correct acidosis by limiting excess protein and prescribing sodium bicarbonate to achieve an alkaline reserve of > 22 mmol/L
		Counteract the usual electrolyte imbalance in uropathy: free sodium diet and supplementation if necessary
		Note that potassium retention is rare in malformative uropathies, allowing normal potassium intake even in advanced stages of renal failure
		In patients with a GFR < 60 mL/min/1.73 m^2^, where growth remains retarded despite nutritional optimization, treatment with growth hormone injection may be indicated
2	Management of anemia to obtain a normal hemoglobin level for age: correction of iron deficiency, folates and if necessary, treatment with erythropoiesis stimulating agents	
3	Management of bone and mineral metabolism disorders: these disorders increase with decreasing GFR and include hyperphosphatemia, hypocalcemia, vitamin D deficiency and hyperparathyroidism. The role of the dietician is essential in reducing phosphate intake and ensuring normal calcium intake. Vitamin D supplementation (ergocalciferol or cholecalciferol) is systematic and adapted to the age of the child with the 25-OH level maintained within recommended targets. When renal insufficiency is advanced, active derivatives of vitamin D (Un-alfa^©^) are used and the addition of phosphorus binders becomes necessary	
4	Cardiovascular prevention: Blood pressure measurement should be part of the clinical examination. The cuff should be appropriate for the size of the child. In CKD, the target blood pressure is in the 50 th percentile of the norms for age and height. In PUV, hypertension is uncommon due to the usual sodium depletion and only occurs in the later stages of renal failure. It may sometimes occur earlier in cases of renal scarring secondary to pyelonephritis. A conversion enzyme inhibitor (CEI) or an angiotensin II antagonist should be used as a first-line treatment, regardless of the level of associated proteinuria, subject to monitoring of the blood chemistry and a cautious and gradual dose increase. In parallel, a healthy lifestyle is recommended with regular physical activity, fighting against obesity and information about the harmful effects of smoking	
5	Preservation of renal function. Avoid UTIs, episodes of dehydration (prompt medical consultation in case of digestive disorders) and nephrotoxic drugs (NSAIDs) or limit their use (aminoglycosides). Adapt drug doses to the level of GFR and check blood tests if available to detect the appearance of pathological microalbuminuria and, if necessary, propose treatment with CEI	
6	Preparation for renal transplantation (RT). In the event of renal failure, renal replacement therapies will inevitably be needed with dialysis and RT at an age that can vary from the first year of life to adulthood, depending on the degree of damage. In any case, it is important to anticipate this deadline from the neonatal period:	
		By preserving the child’s venous and arterial capital as it may be necessary to create an arteriovenous fistula for hemodialysis one day. Arm punctures should be avoided, and blood samples should be taken on the back of the hands. If frequent injections are needed for antibiotic therapy or episodes of dehydration, or in the event of frequent ionic controls, particularly in infants with severe renal insufficiency, an implantable central line catheter should be discussed
		By limiting transfusions as much as possible. This can immunize the child in the HLA system and thus reduce his access to a renal transplant
		Anticipating vaccinations and the specific pre-transplant assessment

### Dietetics

The assistance of a dietician specialized in pediatric nephrology is essential for children with renal failure. The goal of dietary management is to correct growth retardation while limiting the nutrients that can no longer be properly metabolized or eliminated due to renal failure. The dietary proposals must be individualized to the child’s medical status as well as to his preferences. The recommendations below are taken from the NDCP on Chronic Kidney Disease in children [72]. Caloric intake should be at least 100% of the recommended nutrient intake for the age group and then adapted according to the growth in height and weight gain. Nutritional support should be considered when feeding is spontaneously insufficient, in the form of enriching oral intake, or by enteral nutrition if necessary (in newborns or infants, rarely necessary in older children). In this case, a gastrostomy is often preferable to the placement of a gastric tube as the latter is poorly tolerated and if the nutritional support must be prolonged more than a few months. Recommended protein intake ranges from 8 to 13% of normal total energy intake depending on CKD staging. Sodium and water intakes are adapted on a case-by-case basis and are often important until the advanced stages of renal failure, including during dialysis. Vitamins and trace elements intakes should be 100% of the recommended daily allowance. Supplements can be given if intakes are inadequate.

## Urological monitoring in childhood and adolescence

### Bladder function

The aim of this follow-up is to detect and manage any bladder dysfunction related to valve bladder syndrome. Valve bladder syndrome is a complication of PUV, combining dilation of the upper urinary tract, thickening of the bladder wall, and VUR. All these changes can strongly affect the kidneys and lead to bladder dysfunction. The bladder has three functions:

- Aa reservoir function: the accumulation of urine takes place at low pressure, and the bladder must be compliant.
- A storage function, which allows the child to be continent thanks to sphincter resistance.
- An emptying function that must be carried out without post-voiding residual (PVR) and without dyssynergy.

The clinical assessment looks for symptoms of urinary leakage, detrusor-sphincter dyssynergy (DSD), and UTIs. A voiding or CIC schedule is essential to complete this clinical assessment. A child should thus record the amount of fluid intake, the frequency and volume of voiding over a day, and the amount of urine leaks [62]. The bladder function test evolves as the child ages. Some of these examinations require the child’s participation and must be adapted to the clinical progression, age, compliance, and, in some cases, the acquisition of urinary continence (potty training). It includes at least uroflowmetry with electromyography and PVR measurement. Uroflowmetry is used to measure urine volume and flow. The results obtained reflect the proper functioning of detrusor-sphincter coordination, bladder contractility and sphincter relaxation. In combination with an ultrasound examination, PVR measurement can be determined. In practice and according to the ICCS, a PVR > 10% of the estimated bladder capacity for the age is indicative of a bladder voiding disorder [76]. Urodynamic assessment is an examination performed during the follow-up of a child with PUV at defined periods according to the usual protocols. In association with cystography, video-urodynamics (VUDS) is interesting to assess VUR and determine whether this reflux appears at high or low pressure [62]. High pressure phenomena in a low compliant bladder alters the upper urinary tract, aggravates hydronephrosis, reduces the glomerular filtration rate and leads to polyuria and tubulopathy [63, 77]. VUDS is an invasive procedure with a rectal and bladder catheter, perineal electrodes, a slow bladder filling and a voiding when needed. However, this study may explore the myogenic failure of the bladder that appears flaccid and multidiverticular. There are two main categories of “valve bladder”: the overactive high-pressure bladder with low compliance, and the hypoactive and highly compliant bladder (large hypocontractile capacity) with detrusor failure [62]. These two categories can only be distinguished by urodynamic studies. Fifty five percent of children with PUV have bladder dysfunction and 22% have VUR [24]. However, these figures are difficult to estimate, as there are numerous types of bladder dysfunction with different types of management. These figures also change with age. An estimated 66% of children born with PUV under the age of 5 years have an overactive bladder, compared to 15% of those over the age of 13 years. Conversely, PVR increases with age, which is why an uroflowmetry assessment is essential at each consultation, and a VUDS must be repeated throughout the child’s follow-up. The most common symptom of bladder dysfunction is urinary incontinence, which varies between 13 and 70% [24]. In the study by Smith et al*.*, 20 out of 100 patients were continent by 5 years of age, 50 by 10 years, and almost all at 20 years [67].

To delay the onset of ESRD, patients at risk should be monitored and possible bladder dysfunction should be anticipated and actively treated. This urological follow-up should be continued into adulthood in these patients, especially in cases of CIC, with a high risk of non-compliance. A consultation for transitional urology between pediatric and adult urology teams should be encouraged.

In case of persistent bladder dysfunction, several options are available even if not authorized by the French Medication Regulations under the age of 5 (oxybutynin) and 16 years (alfuzosin): anticholinergics, alpha-blockers and CIC. The effectiveness of treatment is monitored by assessment of PVR and upper urinary tract dilation, as well as by UDS. Early use of anticholinergic therapy (oxybutynin) in infants with high voiding pressures and/or low bladder capacity after endoscopic PUV section may have beneficial effects on bladder function and capacity, although comparative studies are needed [68, 77]. However, periodic monitoring is necessary and should be performed after assessment of bladder function and ensuring the absence of significant PVR**.** Alpha-blockers (alfuzosin) can be added to decrease urine outlet pressure and thus promote bladder emptying [62]. If medical treatment is not sufficient, daytime CIC or even at night-time should be offered. CIC is considered in patients with increased PVR, recurrent fUTIs or worsening urinary tract dilation and impaired renal function [3]. This can reduce the intensity of urinary tract dilation, impaired renal function, or impaired bladder function [62]. (Box 3).

**Box 3:** Urological management strategies based on age and bladder function in PUV patients

1	**What should be done for a child over six years of age who is being followed for PUV with renal failure with a video-urodynamic study (VUDS) showing a large, hypercompliant, low-pressure bladder with significant PVR without reflux?** Clinically, the child often reports symptomatic day and night urinary incontinence, with recurrent fUTIs. He has a hypersensitive large bladder capacity and a significant post voiding residual (PVR). Clean-intermittent catheterization (CIC) ensures efficient emptying of the bladder, thus reducing the occurrence of UTIs (caused by a significant and permanent PVR) and providing social continence. This CIC can be obtained through the urethra or by a continent appendicovesicostomy called a Mitrofanoff channel
2	**What should be done for a child under three years of age who is being followed for PUV with renal failure with a VUDS showing a hypoactive, high-pressure overactive bladder?** This is often a child who is always in nappies, who may have UTIs, and who is therefore at greater risk of renal damage. Only a VUDS can determine this risk. This child will require anticholinergic and CIC. This patient will need to be monitored every three months, with repeated CIC diaries and check-ups. If this first -line treatment fails, other procedures should be discussed, including BA (with the ureter if the kidney is not functional or with the ileum, an indication which remains rare). A VUDS should be systematically and repeatedly performed in children with PUV to assess compliance, the existence of high-grade high-pressure reflux, hyperactivity, significant PVR, and the measurement of the bladder contractility index (BCI). This index divides bladder contractility into 3 groups: strong (BCI > 150), normal (100 < BCI < 150) and weak (BCI < 100). It is calculated with the following formula: BCI = P_det_*Q_max_ + 5 Q_max_, where Q_max_ is the maximum urine flow, and P_det_ is the detrusor pressure

Finally, in some rare cases where the child has anuria (following bilateral ureterostomy, anuric CKD), it may be necessary to use temporary continuous bladder irrigation via a suprapubic catheter to assess bladder function before renal transplantation (RT). This will be considered to avoid an urgent bladder augmentation after transplantation.

### Fertility

Fertility problems have been reported in patients with PUV, but no studies including a large number of patients have been performed. The actual incidence of these disorders is currently unknown. Not all patients are infertile, some may have children, and the cause of infertility appears to be multifactorial. Other patients will present with retrograde ejaculation [69]. In the study by Woodhouse et al*.* of 21 patients with PUV, half had no ejaculation problems and one third reported erectile dysfunction [78].

## Urinary tract infection and its prevention

Recurrent febrile urinary tract infections (fUTIs) are common in patients with PUV and are mainly related to VUR and bladder dysfunction. The combination of long-term antibiotic prophylaxis with optimal management of the “valve bladder syndrome” can decrease the prevalence of UTIs in these children [79]. Circumcision and early diagnosis in the antenatal period appear to be factors that decrease the risk of UTIs. The risk of UTI was reduced by 92% in circumcised patients [80]. This result is confirmed by the only published multicenter randomized clinical trial. In the “CIRCUP” RCT study of 91 patients followed for two years in France, the risk of developing a UTI was 20% in patients receiving antibiotic prophylaxis alone, compared with 3% in patients with combined antibiotic prophylaxis and circumcision at birth. In this French study, uncircumcised children were 10.3 times more likely to develop a UTI than children who had had a circumcision [81]. In another study, children diagnosed after birth had a 6.5-fold higher risk of developing UTIs than patients with an antenatal diagnosis. This difference is explained by 1) early antibiotic prophylaxis in these children 2) early surgery 3) regular multidisciplinary follow-up [82].

## Management of stage V renal failure

Stage V renal failure (formerly end-stage renal disease [ESRD]) is common in cases of bilateral upper urinary tract involvement or in cases of PUV associated with a single kidney. Overall, despite many advances in prenatal diagnosis and intervention, as well as in early postnatal assessment and treatment, the prevalence of ESRD in boys with PUV is between 20 and 50% [3]. The estimated cumulative incidence of stage V CKD in patients with PUV is 10% at 5 years, 15% at 10 years and 31% at 18 years. This change in incidence is related to the progression of renal dysplasia lesions [83]. The incidence of proteinuria and hypertension is not negligible in these patients. They are strong markers of impaired renal function. It is therefore advisable to check blood pressure, proteinuria and GFR in these patients [82]. Preservation of renal function and optimal renal development are the main goals in the management of patients with PUV. Renal damage in patients with PUV is a consequence of dysplasia and obstruction (Box 4).

**Box 4:** Recommendations of French national diagnostic and care protocol (NDCP) for posterior urethral valves (PUV)

Recommendation 1	There are no studies defining the likelihood of or diagnostic scores of ultrasound criteria for PUV. When PUV is suspected, the probability of the diagnosis is determined based on the ultrasound findings during a multidisciplinary discussion, then perinatal management is proposed	
Recommendation 2	These three criteria, when present, strongly suggest a progression to fetal renal failure. When none of these criteria is present on ultrasound, the prognosis is usually more reassuring but does not exclude renal failure in childhood. Finally, it is important to remember that sequential screening of these ultrasound parameters must be performed as they may develop one after the other in these fetuses. In the most complex situations, the ultrasound picture is incomplete with the presence of only one or two criteria, making prenatal advice particularly difficult. Second line investigations must be performed in these cases, in particular fetal biochemistry to obtain further prognostic information	
Recommendation 3	These prenatal biochemical tests improve the advice given to parents after assessing the renal prognosis of their unborn child. The two types of samples (urine or blood) provide different information. The choice of test is made according to the gestational age and the presentation of the fetus. Fetal urine can also be used if urodigestive fistulae are suspected. The differential diagnosis with MMIHS is very interesting in cases where the US give no clear information. The laboratory should be provided with fetal urine and amniotic fluid for this specific diagnosis. Because of their invasiveness these tests are often only indicated in cases with signs of severity on ultrasound. Strict indications are listed as below:	
		Poor progression of ultrasound features
		Assessment before possible in utero treatment
		Assessment before possible termination of pregnancy when a poor prognosis is suspected
Recommendation 4	*Patients with a fetus presenting with LUTO should be referred to an expert prenatal diagnostic center*. The evaluation will confirm or infirm the diagnosis of LUTO and the causes (including PUV), search for associated morphological and chromosomal abnormalities, and provide a prognostic assessment. A multidisciplinary consultation including neonatologists, pediatric urologists and pediatric nephrologists should be offered when PUV is suspected. Prenatal therapeutic management may be considered, presenting the risks associated with the procedure and the uncertainty of the expected benefit, particularly in terms of renal function. The patient may request termination of pregnancy, which should then be discussed at the Multidisciplinary Center for Prenatal Diagnosis. Finally, a “wait and see” option can be offered. If possible, the birth should be organized in a level 3 maternity hospital with an onsite pediatric urologist and a pediatric nephrologist to provide immediate multidisciplinary neonatal care	
Recommendation 5	*Early endoscopic intervention* Perform neonatal endoscopic valves ablation within 48 h after birth for clinically stable newborns. Use a cold knife for the valve section to minimize the risk of postoperative urethral strictures; Utilize miniaturized endoscopic instruments (< 2 mm) for newborns weighing more than 2000 g *Management of low birth weight infants* For newborns weighing less than 2000 g, prioritize bladder drainage through diversion techniques; Consider vesicostomy as the preferred temporary diversion to preserve renal function until the infant is stable enough for valve section *Temporary urinary diversions* Use Sober’s cutaneous ureterostomy in cases where vesicostomy is unsuccessful or as an alternative. This approach preserves part of the bladder function; Reserve pyelostomy/nephrostomy for cases complicated by urinary tract infection or pyonephrosis as a temporary measure	
Recommendation 6	Two algorithms for the management of PUV at birth and at follow-up are proposed in Fig. 2 and 3 respectively	
Recommendation 7	(suggested frequency based on expert experience and current practice, to be adapted according to clinical course)	
		Renal and bladder ultrasound: one month after the endoscopic resection and then every three months for the first year, then annually
		Post-operative control VCUG: to be performed according to the clinical progression and ultrasound results
		DMSA scan: at 6 months
Recommendations 8	*Regular bladder function assessments* Conduct routine assessments of bladder function, including uroflowmetry with electromyography and post-void residual (PVR) measurements, at each follow-up visit; Perform urodynamic studies at defined intervals to monitor bladder compliance and detrusor function, distinguishing between overactive high-pressure bladders and hypoactive large-capacity bladders	
	*Management of bladder dysfunction* Initiate early use of anticholinergic therapy, such as oxybutynin, in infants with high voiding pressures and/or low bladder capacity post-endoscopic PUV section to improve bladder function and capacity; Consider adding alpha-blockers (e.g., alfuzosin) to decrease outlet resistance and promote bladder emptying if anticholinergic therapy alone is insufficient	
	*Clean Intermittent Catheterization (CIC)* Introduce CIC in children with significant PVR, recurrent febrile urinary tract infections (fUTIs), or worsening upper urinary tract dilation and renal function impairment; Educate families on proper CIC techniques, ensuring they understand the importance of regular catheterization to prevent urinary retention and associated complications	
	*Monitoring and management of urinary tract infections (UTIs)* Implement prophylactic measures, including antibiotic prophylaxis and circumcision, to reduce the incidence of recurrent fUTIs; Provide guidance on early recognition and treatment of UTIs to prevent renal damage	
	*Long-term follow-up* Schedule regular follow-up visits to monitor renal function, bladder dynamics, and overall growth and development; Include routine renal and bladder ultrasounds, initially performed monthly and then at longer intervals based on clinical progress, to assess the progression of urinary tract dilation and bladder wall changes	
	*Transitional care to adult services* Plan for a structured transition from pediatric to adult urology and nephrology services, ensuring continuous and consistent care into adulthood; Facilitate communication between pediatric and adult care teams to maintain comprehensive management of bladder dysfunction and renal health	
	*Patient and family education* Provide ongoing education to patients and their families regarding the importance of adherence to follow-up schedules, medication compliance, and the management of bladder dysfunction; Offer psychological support and counseling to address the emotional and social challenges associated with long-term bladder management and potential renal complications	
	*Use of imaging in follow-up* Perform annual renal and bladder ultrasounds to monitor kidney growth, detect new cortical scars, and assess bladder wall thickness and regularity; Utilize dimercaptosuccinic acid (DMSA) renal scintigraphy to evaluate relative renal function and search for cortical scars, providing prognostic information	
Recommendations 9	Managing Stage V renal failure in children with posterior urethral valves (PUV) involves early identification, continuous monitoring, and a multidisciplinary approach. Regular assessments of renal function, including serum creatinine, blood urea nitrogen, electrolytes, blood pressure, and proteinuria, are essential. A team of pediatric nephrologists, urologists, dieticians, and psychologists provides comprehensive care and supports families through education and counseling	
	Preparation for dialysis should prioritize peritoneal dialysis for younger children, with necessary procedures like gastrostomy performed beforehand to minimize infection risks. Nutritional management is crucial to support growth and maintain fluid and electrolyte balance, utilizing specialized formulas with reduced phosphorus and potassium. Psychosocial support is vital to help families cope with the emotional and social challenges of long-term dialysis	
	Pre-emptive renal transplantation (RT) is preferable to avoid prolonged dialysis, with bladder preparation starting years in advance. Regular urodynamic assessments are necessary to manage bladder dysfunction effectively. Post-transplantation follow-up ensures monitoring for urological complications, adherence to immunosuppressive therapy, and long-term graft survival	
	Addressing complications such as growth retardation, anemia, metabolic bone disease, and cardiovascular health through appropriate medical and dietary interventions is essential. Implementing these recommendations improves outcomes and quality of life for children with Stage V renal failure due to PUV, ensuring a structured and comprehensive approach to their care	
	By following these guidelines, healthcare providers can significantly improve the prognosis and overall well-being of patients with Stage V renal failure caused by PUV, ultimately leading to better long-term health outcomes and quality of life	
Recommendation 10	The management of CKD is multidisciplinary and includes the paramedical team (social worker, nurse specialized in patient education, dietician, clinical psychologist) who are familiar with CKD. Psychological support for the child and the family is an essential aspect for the balanced development of a child who is subject to these constraints	
Recommendation 11	Medical and social care is discussed after the patient’s situation has been assessed and medical and family information has been obtained. Several points are taken into account:	
		Ensure that a care protocol is in place
		When the child’s state of health requires the presence of a parent for compulsory care, a parental presence leave may be negotiated with the employer with a daily parental presence allowance
		Possibility of setting up a specific file to: i) recognize the disability; ii) payment of an allowance; iii) support needs at school; iv) educational and medico-social referrals; and v) professional orientations

Renal dysplasia is the most important factor and determines long-term outcomes [3]. It is irreversible and lead to a decrease the growth and development of the kidneys. A prenatal diagnosis helps to identify newborns at risk of renal failure. Optimal management of fUTI and bladder dysfunction can reduce and delay renal function impairment [84]. The second factor for progression to ESRD in children is the obstructive nature of the uropathy (16.3% of children with PUV require renal transplantation (RT)) [3]. Proteinuria and hypertension are known factors in the progression of renal failure. They may appear over time, especially in the second decade. It is advisable to monitor blood pressure and proteinuria in these patients [82].

### Dialysis

Despite regular and strict follow-up, about one third of patients with PUV will progress to stage V CKD in childhood [83]. Indeed, renal failure may be stabilized in childhood, but due to nephron reduction, it will most often worsen in adolescence or young adulthood. In stage V renal failure, if RT could not be preemptive and dialysis is necessary, both modalities of dialysis (peritoneal dialysis or hemodialysis) can be offered depending on the preferences of the child and his family. There are no contraindications to peritoneal dialysis, including gastrostomy or the presence of a Mitrofanoff channel for CIC, if care is taken to place the emergence of the dialysis catheter at a distance from these stomas. However, the gastrostomy should be performed before starting peritoneal dialysis, if possible, to reduce the risk of infection. Despite the development of specific new hemodialyzers (Carpediem^©^, Medtronic and Nidus^©^, Fresenius), for very young infants, and neonates, peritoneal dialysis is the preferred method due to the difficulties of vascular access in case of hemodialysis and the miniaturization of extracorporeal circuits. In some neonates, dialysis may only be temporary because renal function may improve after the endoscopic valve ablation and thanks to the physiological maturation of glomerular filtration in the first year of life [85]. In most cases, the possibility of neonatal dialysis can be anticipated by antenatal data (anamnios, ultrasound appearance of the renal parenchyma, 2-microglobulin). Alike respiratory support must be discussed with the parents, neonatal dialysis should be explained as objectively as possible (possibilities and limits of the techniques, their consequences on the child’s quality of life and development, as well as their indispensable involvement in the medical care of their child). In these difficult cases, it is important that the parents meet with a multidisciplinary team before the birth of the child to discuss the different therapeutic options well in advance of their implementation. It should be noted that if conservative treatment of renal failure is well conducted, dialysis is never necessary in the first few days of life, even in the case of anuria, and can usually be delayed until the end of the first week of life, giving the parents’ time to reflect.

### Renal transplantation: bladder preparation before renal transplantation

Renal transplantation (RT) is preferable to dialysis because of the advantages to the quality of life and it should be performed pre-emptively if possible, before the need for renal replacement therapy [86]. Bladder preparation should be planned years in advance. The lower urinary tract is markedly altered in patients with PUV, and urological complications such as hematuria, UTIs, urethral strictures and fistulas, stones and urinary retention are more common in this population [2]. The incidence of these complications after RT varies from 1 to 15% according to studies [87]. Before the patient can be registered on the renal transplant list, several clinical, radiological, functional, and biological assessments must be performed according to an established protocol including an evaluation of the bladder function by the pediatric urologist on the renal transplantation team the child has been assigned to. A full evaluation must be performed for optimal preparation of the bladder before RT, including repeated (video-)urodynamics, to diagnose bladder dysfunction and to define the best management. This preparation is managed by a multidisciplinary team with a nurse specializes in patient education and a psychologist working closely with the child and the parents. These patients must be regularly seen and followed up for regular consultations with a voiding or CIC diary, uroflowmetry, renal and bladder ultrasound, annual VUDS and biological blood and urine tests with a pediatric nephrologist. Regular communication and information between the nephrologists and the pediatric urologists are needed to share the surgical management**.** Bladder dysfunction is a prognostic factor for RT failure. Graft survival in patients with PUV and bladder dysfunction at 1, 3 and 5 years is 88–90%, 83–90% and 65–76%, respectively [87, 88]. After 10 years of follow-up, this rate varies from 54 to 77.3% depending on the study [88]. After 15 years of follow-up, this survival is significantly reduced in patients with bladder dysfunction compared to patients without (70% versus 85%, respectively). This difference can be explained by a higher rate of pyelonephritis in patients with “valve bladder syndrome”, and fUTI exposes the patient to a risk of renal cortical graft scarring which is a prognostic factor for graft loss [87]. There are several options to manage bladder function before RT, including the use of anticholinergics, CIC and sometimes bladder augmentation (BA). The latter is still a subject of debate, and no recommendations have been made. The indications for BA are poorly defined and include a non-compliant bladder with low capacity and detrusor overactivity despite medical treatment. However, the use of BA is not always necessary in children with PUV. Almost 50% of children do not show signs of bladder dysfunction after RT, and therefore do not require BA [89, 90]. Nevertheless, most studies agree that there is a higher rate of infection in patients with PUV after RT when BA is done before RT [86, 89, 91–94]. In recent years, the mortality rate of children born with PUV has decreased dramatically, from 20% in the 1960’s to less than 5% today. In today’s modern healthcare system, the mortality rate is 1%. Recent reports indicate a perinatal mortality rate of 120 to 458 per 1000 births for the most severe cases [10]. However, this rate is probably underestimated due to termination of pregnancy for severe cases diagnosed prenatally, and cases of fetuses dying in utero [30]. In addition, the decrease in mortality has probably contributed to the increase in renal function impairment in children and adults (increase in CKD and ESRD rates of 37.5% and 22.5%, respectively in the study by Vasconcelos et al*.* [83]. Death is not related to the time of diagnosis. Earlier diagnosis improved respiratory support at birth, and the current management of impaired renal function in newborns and children have probably played an important role in improving survival rate. However, these improvements have not resulted in increased long-term survival rate [80, 82].

## Psychological care and follow-up

Marokakis et al*.* studied the psychological impact of the announcement of a prenatal diagnosis of CAKUT on parents. Most parents reported that they needed information about their child’s care and treatment options. These families did not hesitate to contact a support group of parents in their same situation. The continuity of care from the time of prenatal diagnosis makes it possible to build a secure link between parents and clinicians. However, access to psychological support after the birth is needed [95]. In a study about 20 boys published in 2020, Monteiro et al*.* report that most children (85%) born with PUV do not show psychomotor delay. Nevertheless, neurodevelopmental delays increase with the severity of the obstruction and are even more present in patients with dialysis [96]. Jalkanen et al*.* investigated the quality of life of 108 Swedish patients who had surgery for PUV during childhood. These patients reported a good quality of life, and their scores were even better than those of the general Swedish population. Although most people with PUV live well, without any symptoms, some patients experience a decreased quality of life due to renal failure and/or urinary incontinence. Psychological support for these patients is necessary. Incontinent patients seem to suffer the most from this condition and tend to sleep less and have lower sexual activity. These patients are more likely to urinate at night than the general population, which explains the sleep disturbances. In addition, they tend to have difficulties in experiencing pleasure and achieving orgasm. These disorders are absent in continent patients [97]. Swedish researchers have studied the determinants of good quality of life in seven patients with PUV who used CIC. These children, aged between 6 and 16 years, made a point of feeling respected and setting limits on what they could and could not accept from their friends. They were concerned about disclosing their daily treatment, including the use of CIC. Daily treatments need to be well planned during the day, depending on activities. When intermittent catheterization is well accepted by the child, life seems easier to live. Acceptance helps the child to have regular, non-negotiable daily routine. Despite this, the degree of acceptance changes over time and between individuals. Simple events, such as a lack of toilet, is enough to stop catheterization. Easy hospital visits are essential to the child’s well-being. The child accepts his/her illness better when s/he is aware of the planned examinations and understands his diagnosis and what it involves. Children also need to get to know the nurses and clinicians who care for them to create a strong bond of trust [98]. Children adopt several strategies to cope with problems and difficulties: they practice positive thinking (“you can do it”, “it could be worse”, “if you do it often you will get used to it” …) or think of something pleasant they can do after an unpleasant treatment.

## Therapeutic education of patients

Therapeutic education should focus on the management of voiding habits and clean intermittent catheterization (CIC), and the management of renal failure. When the child is old enough, he should keep a daily voiding diary, recording the number of urinations, the amount of urine, the amount of fluid taken, and the number and grade of leaking. The parents of children on CIC must learn how to perform this daytime and sometimes at nighttime procedure independently. The procedures must be clearly explained by a specialized nurse in patient education. Finally, families should be informed of the risk of renal failure and how to manage it. As described above, it may be necessary to train parents in enteral feeding techniques using a nasogastric tube or a gastrostomy. As renal failure progresses, children and parents will be included in specific programs to prepare for RT, and if this cannot be done pre-emptively, in programs to prepare for dialysis, whatever the technique chosen [95–98].

## Conclusions

The French National Diagnostic and Care Protocol for posterior urethral valves represent a critical advancement in the comprehensive management of this congenital anomaly, which significantly impacts renal function and overall health from the fetal stage through adolescence. As the leading cause of lower urinary tract obstruction in children, posterior urethral valves poses a severe prognosis, with a substantial proportion of affected individuals progressing to end-stage renal disease.

These guidelines underscore the necessity of early and accurate prenatal diagnosis through advanced imaging techniques, coupled with a coordinated multidisciplinary approach that includes pediatric urologists, nephrologists, and other relevant healthcare professionals. By delineating standardized diagnostic and therapeutic guidelines, the French National Diagnostic and Care Protocol aim to optimize outcomes through timely interventions such as vesico-amniotic shunting and endoscopic valve ablation, which are pivotal in alleviating obstruction and preserving renal function.

Long-term management, as highlighted by these guidelines, is essential for monitoring renal function, addressing bladder dysfunction, and preventing recurrent urinary tract infections and chronic kidney disease. The guidelines advocate for continuous, regular follow-up and the involvement of a multidisciplinary care team to ensure the best possible patient outcomes and quality of life.

By harmonizing care practices and identifying essential off-label pharmaceuticals and services, the guidelines strive to provide consistent, high-quality care across healthcare settings in France. This structured approach aims not only to reduce the morbidity associated with posterior urethral valves but also to enhance the long-term prognosis for affected children, thereby setting a benchmark for the management of this rare yet impactful condition in pediatric urology.

## Data Availability

The data that support the findings of this study are not publicly available due to privacy reasons but are available from the corresponding author upon reasonable request.

## Declarations

### Ai statement

Generative AI and AI-assisted technologies were NOT used in the preparation of this work.
